# A rare case of non-oncologic metastasis: Keep calm and check the veins

**DOI:** 10.1016/j.intf.2025.100342

**Published:** 2026-01-03

**Authors:** Imane Habbal, Francisca Joly, Raphael Bacquet, Eric Le Bihan, Flore de Castelbajac

**Affiliations:** Université de Paris, Assistance Nutritive, Hôpital Beaujon APHP, Paris, France

**Keywords:** Parenteral nutrition, Catheter, Intestinal failure, GLP-2 analogs, Venous thrombosis

## Abstract

We report the case of a 57-year-old patient with short bowel syndrome and chronic intestinal failure secondary to multiple resections for Crohn’s disease, requiring parenteral nutrition (PN) since 1997. During an annual follow-up, the patient reported difficulties with his central venous access. Computed tomography (CT) revealed pseudo-sclerotic lesions of the T1-T3 vertebrae, suggestive of metastatic disease. Further imaging showed severe stenosis of the superior vena cava related to chronic thrombosis of the central venous line. Endovascular dilatation of the stenosed vein led to complete resolution of the lesions. This phenomenon, known as “vanishing bone metastases” (VBM), results from contrast reflux into collateral venous pathways, and can mimic bone metastases. Lack of recognition of this rare but significant radiological artifact may lead to unnecessary invasive procedures. Awareness of VBM is crucial, especially in patients on long-term PR who are at high risk of thrombosis, to prevent misdiagnosis and improve patient care.

We report the case of a 57-year-old patient with short bowel syndrome and chronic intestinal failure secondary to multiple resections for Crohn’s disease. Parenteral nutrition (PN) was initiated in 1997 due to intestinal failure. The last central line, a single lumen catheter, was inserted in 2015. Prior to this, the patient had already required long-term central venous access, exclusively through central lines. The patient was treated with GLP-2 analogs for 4 years with a significant benefit, reducing PN requirements from seven days per week to five days per week, but without weaning off PN. TauroLock was used as the catheter lock solution and the patient did not experience any central line associated bloodstream infections during this period. During the annual routine follow-up, the patient reported having some difficulties with the central line for a few weeks. Computed tomography (CT) revealed pseudo-sclerotic bone lesions, hyperdense in the portal phase, involving the vertebral bodies and pedicles from T1 to T3 **(**Fig. 1a). The radiological findings initially suggested a metastatic disease. However, the radiologists noted severe stenosis at the origin of the superior vena cava, associated with chronic thrombosis of the central venous line. The thoracic veins play a key role in venous drainage of the upper trunk (1). The obstruction of these veins is mostly acquired, usually due to thrombosis. In 80 % of cases, malignancies are the underlying cause, but iatrogenic factors such as catheter insertion may also contribute (2). Catheter-related central vein thrombosis is a severe complication of home (PN) that may be clinically manifest or subclinical (3). The patient was already receiving anticoagulation therapy for atrial fibrillation, however thrombolysis was not feasible. Given these considerations, we decided to perform endovascular dilation of the stenosed vein when inserting a new central line. A follow-up CT scan showed the complete resolution of the bone lesions previously observed (Fig. 1b).

**Fig. 1a:**
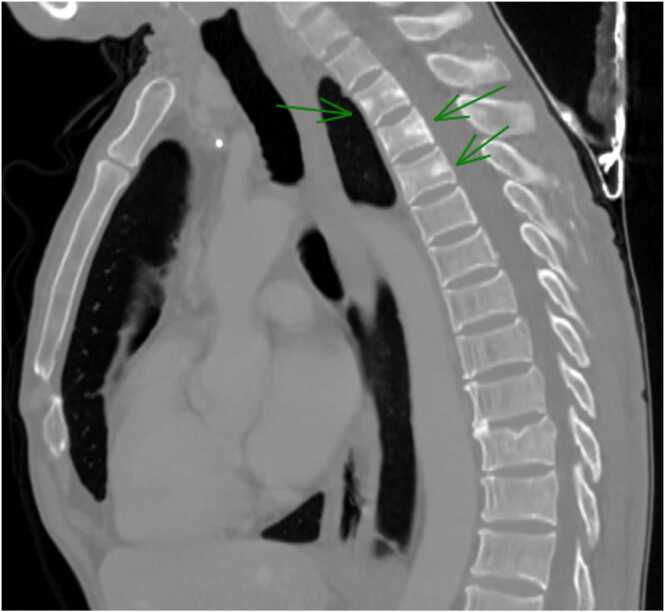
Sagittal section of a contrast-enhanced CT scan showing pseudo-sclerotic bone lesions, hyperdense in the portal phase, involving the vertebral bodies and pedicles from T1 to T3.

**Fig. 1b:**
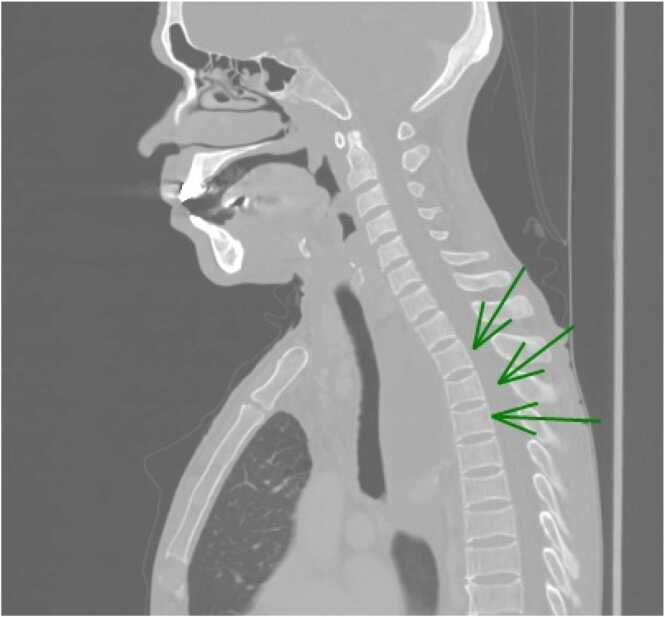
Sagittal section of a contrast-enhanced CT scan after dilation of the stenosed vein showing the complete resolution of the bone lesions previously observed.

In case of chronic venous occlusion, collateral pathways develop to maintain venous drainage (4). The phenomenon referred to as “Vanishing Bone Metastases” (VBM) is associated with contrast material reflux into these collateral pathways, particularly into the paravertebral venous plexus, also known as Batson’s venous plexus **(**Fig. 2) (1).

**Fig. 2:**
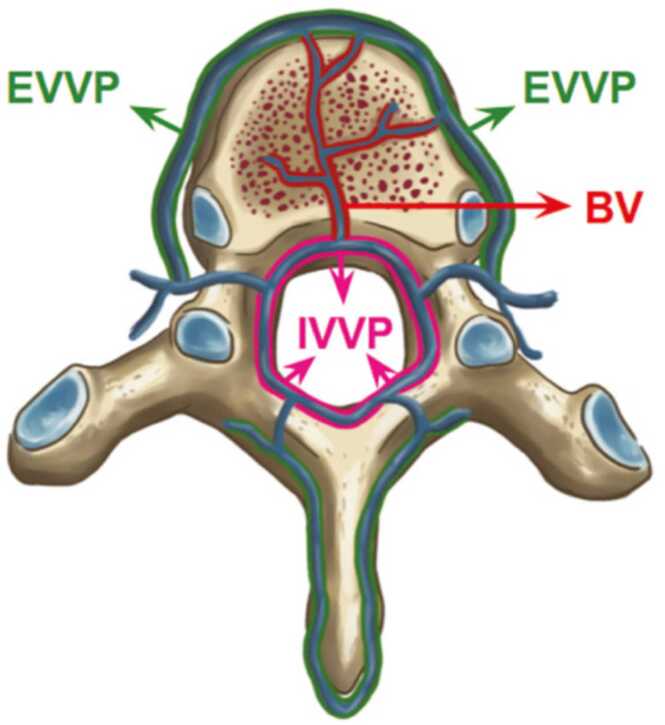
Axial section of a vertebra showing the basivertebral vein (BV), external vertebral venous plexus (EVVP), and internal vertebral venous plexus (IVVP).

This case highlighted the importance of recognizing VBM, a rare but significant imaging artifact that may mimic a metastatic disease. Although sporadic cases have been reported in the literature (5), none has been specifically related to long-term PN. Awareness of this phenomenon is crucial, especially in oncology, where patients frequently receive PN and are at high risk of thrombosis. Misinterpretation of imaging findings could lead to unnecessary procedures, such as bone biopsies, with potential complications.

Furthermore, the term “Vanishing Bone Metastases” may be misleading, as it suggests both the presence of a malignancy and a lesion that is disappearing, neither of which is accurate. Reconsidering its nomenclature could be warranted to prevent any undue concern among clinicians. This case also highlighted the need for vigilance when diagnosing bone lesions in patients with chronic venous obstruction. Increased awareness of VBM could prevent misdiagnosis and unnecessary interventions, thereby improving patient care.

## CRediT authorship contribution statement

**Francisca Joly:** Supervision. **Eric Le Bihan:** Resources. **Raphael Bacquet:** Visualization. **Flore de Castelbajac:** Conceptualization. **HABBAL Imane:** Writing – original draft.

## Ethical clearance

Not required

## Patient's consent

A non-opposition statement was obtained from the patient for publication of this case report.

## Funding

This research did not receive any specific grant from funding agencies in the public, commercial or not-for-profit sectors.

## Declaration of Competing Interest

The authors declare that they have no known competing financial interests or personal relationships that could have appeared to influence the work reported in this paper.

